# Effectiveness and Cost-effectiveness of Minimal Ovarian Stimulation *in-vitro* Fertilization versus Conventional Ovarian Stimulation in Poor Responders: Economic Evaluation Alongside a Propensity Score Adjusted Prospective Observational Study

**DOI:** 10.5935/1518-0557.20220025

**Published:** 2023

**Authors:** Tatiane Oliveira de Souza, Ângela Jornada Ben, Johanna M. van Dongen, Judith E. Bosmans, João Sabino Lahorgue da Cunha-Filho

**Affiliations:** 1 Graduate Program in Gynecology and Obstetrics, Faculty of Medicine, Universidade Federal do Rio Grande do Sul, Ramiro Barcelos 2400, 90035-003 Porto Alegre, Brazil; 2 Department of Health Sciences, Faculty of Science, Vrije Universiteit Amsterdam, Amsterdam Public Health research institute, Van der Boechorststraat 7, 1081 BT Amsterdam, The Netherlands; 3 Department of Gynecology and Obstetrics, Faculty of Medicine, Universidade Federal do Rio Grande do Sul, Ramiro Barcelos 2400, 90035-003 Porto Alegre, Brazil

**Keywords:** *in vitro* fertilization, cost-benefit analysis, poor ovarian response

## Abstract

**Objective:**

Information on the pregnancy rate after successive in-vitro fertilization (IVF) cycles and their associated costs is relevant for couples undergoing assisted reproduction treatments (ARTs). This study, therefore, sought to investigate the effectiveness and the cost-effectiveness of two ARTs, the minimal ovarian stimulation IVF (MS-IVF) compared to the conventional ovarian stimulation IVF (C-IVF) from the payer’s perspective.

**Methods:**

A 10-months follow-up prospective observational study was conducted in a sample of couples who sought ARTs in a private clinic in Southern Brazil. Women had to satisfy the Bologna Criteria and be older than 35 years. The effect outcome was pregnancy rate per initiated cycle. Medication costs were based on medical records. Costs and effect differences were estimated using seemingly unrelated regressions adjusted for the propensity score estimated based on women’s characteristics.

**Results:**

All 84 eligible women who agreed to participate received a total of 92 IVF cycles (MS-IVF, n=27[35 cycles]; C-IVF n=57[57 cycles]. The effect difference between MS-IVF and C-IVF was -5.1% (95%CI, -13.2 to 5.2). Medication costs of MS-IVF were significantly lower than C-IVF by €-1260 (95%CI, -1401 to -1118). The probabilities of MS-IVF being cost-effective compared to C-IVF ranged from 1 to 0.76 for willingness-to-pay of €0 to €15,000 per established pregnancy, respectively.

**Conclusions:**

Even though there were no positive effect differences between groups, MS-IVF might be cost-effective compared to C-IVF from the payer’s perspective due to its relatively large cost savings compared to C-IVF. However, further investigation is needed to confirm these findings in a larger sample.

## INTRODUCTION

Worldwide, infertility affects approximately 10-15% of couples at reproductive age, with an increasing trend in the last decades, especially in low- and middle-income countries (Sun *et al.*, 2019; Zegers-Hochschild *et al.*, 2019). Infertility has been found to be related to the postponement of maternity to an older age, when a natural decline in fertility occurs (Schmidt *et al.*, 2012). This has led to an increased demand for assisted reproduction treatments, such as in-vitro fertilization (IVF). However, assisted reproduction treatments can be relatively expensive and even unaffordable for couples, particularly because IVF treatment protocols are not covered by health insurance companies and/or public healthcare in many countries (Dyer & Patel, 2012; Stephen *et al.*, 2016).

Among assisted reproduction treatments, controlled ovarian hyperstimulation in combination with IVF(i.e., conventional IVF, C-IVF) has been the ultimate treatment option for women with a suboptimal follicular response to ovarian stimulation in combination with IVF (Farquhar & Marjoribanks, 2018; Zhang *et al.*, 2020). These so-called poor responders represent more than one third of women undergoing assisted reproductive treatments (Oehninger, 2011; Zhang *et al.*, 2020). The C-IVF treatment protocol consists of ovarian hyperstimulation with high hormone doses to generate a higher number of oocytes and to maximize the number of embryos available for transfer into the uterus (Shrestha *et al.*, 2015; Nargund *et al.*, 2017). However, these high hormone doses may result in early drop-outs due to adverse effects and an increased risk of multiple pregnancies in combination with their associated complications and high costs (Nargund *et al.*, 2017). In addition, studies have shown that exposure to supraphysiologic hormone levels might be associated with high rates of low birth weight (Shrestha *et al.*, 2015; Nargund *et al.*, 2017). Therefore, alternative IVF treatment protocols have been developed to minimize these unfavourable outcomes (Shrestha *et al.*, 2015).

Minimal ovarian stimulation IVF (MS-IVF), in which lower doses of hormones are taken for a shorter duration, has become increasingly popular because it has been found to create a more natural physiological response (i.e., a hormonal milieu more similar to a natural cycle), with lower levels of discomfort and costs (Nargund & Frydman, 2007; Verberg *et al.*, 2009; Shrestha *et al.*, 2015; Nargund *et al.*, 2017). Lower levels of discomfort may prevent drop-outs, whereas lower costs may allow patients to undergo more treatment cycles for the same amount of money (Eijkemans *et al.*, 2006). This might also be preferred by some couples if possible differences in pregnancy rates are acceptable (Nargund & Frydman, 2007). However, information on the effectiveness and cost-effectiveness of MS-IFV compared with C-IVF is lacking. Therefore, the aim of this study is twofold. First, it aimed to assess whether MS-IVF is effective compared to C-IVF in terms of their resulting pregnancy rates in poor responders. Second, it aimed to evaluate the cost-effectiveness of the MS-IVF compared to C-IVF in poor responders from a payer’s perspective.

## MATERIAL AND METHODS

### Study design

A prospective observational study was conducted comparing the effectiveness and cost-effectiveness of MS-IVF to C-IVF in a convenience sample of couples who sought assisted reproduction treatments at the Centro de Reprodução Insemine in Porto Alegre, Southern Brazil. All couples who sought treatment between December 2016 and September 2017 were assessed for eligibility by the medical team. Couples who satisfied the inclusion criteria were invited to participate. Those who agreed to participate signed an informed consent form. The study was approved by the Research Ethics Committee at Hospital de Clínicas de Porto Alegre (registration number 2016-0410). The economic evaluation follows the good practices for real-world data studies (Berger *et al.*, 2017) and is reported according to the Consolidated Health Economic Evaluation Reporting Standards (CHEERS) statement (Husereau *et al.*, 2013).

### Study population

The study population included women with a suboptimal follicular response to previous ovarian stimulation in combination with IVF, further referred to as poor responders (Oehninger, 2011; Oudendijk *et al.*, 2012; Zhang *et al.*, 2020). Women were considered poor responders based on the Bologna Criteria (Ferraretti *et al.*, 2011), meaning that they had to meet the following criteria: (i) advanced maternal age (≥40 years) or any other risk factor for a poor ovarian response; (ii) a previous poor ovarian response (≤3 oocytes with the C-IVF treatment protocol), and (iii) an abnormal ovarian reserve test (i.e., anti-Müllerian hormone [AMH] below 1.1ng/mL). Women who were classified as poor responders by the medical team were eligible and were invited to participate. Women were excluded if they were 35 years or younger, because fertility drops more rapidly after the age of 35.

### Setting and location

Currently, the Brazilian Public Healthcare System (SUS) and some healthcare insurance companies reimburse infertility-related diagnostic procedures (e.g. laboratory tests, ultrasonography, laparoscopy, and hysterosalpingography), but not IVF treatment protocols (Corrêa & Loyola, 2015). As a consequence, 95% of IVF treatment protocols in Brazil are provided by private clinics and patients need to pay for those treatments themselves (i.e., out-of-pocket costs) (Souza, 2014; Duarte-Filho *et al.*, 2019). One of these private clinics is the Centro de Reprodução Insemine that has a team of specialists in assisted reproductive treatments providing care in Porto Alegre, Southern Brazil, for the last 15 years.

### Study perspective and time horizon

Given the fact that couples need to pay for IVF treatment protocols by themselves in Brazil, this economic evaluation is performed from their perspective (i.e., the payer’s perspective). This means that only costs related to the treatments incurred by couples themselves are included in the analysis. Couples were followed-up for a maximum of 10 months. Therefore, discounting of costs and effects was not necessary (Drummond *et al.*, 2005).

### Treatment protocols

Worldwide, the most frequently used treatment protocol for treating poor responders is C-IVF (Song *et al.*, 2014; Zhang *et al.*, 2020). At the Centro de Reprodução Insemine, both MS-IVF and C-IVF were provided. Couples and medical doctors decided together which treatment protocol couples received based on previous response to ovarian stimulation, and advantages and disadvantages of each protocol. In both groups, a vaginal dose of micronized progesterone (600mg/day) was prescribed during the luteal phase before embryo transfer to prepare the endometrium for embryo implantation. The IVF was performed in a specialized laboratory by either mixing sperm and oocytes and incubating them overnight or by intracytoplasmic sperm injection into oocytes (ICSI). Embryos were transferred to the uterus three days after IVF.

One cycle of either treatment protocol lasted approximately 30 days from the starting day until the pregnancy test. Couples may opt to undergo more than one cycle and/or receive both treatment protocols consecutively under medical advice.

#### Conventional ovarian stimulation in-vitro fertilization - control group

One cycle of the C-IVF treatment protocol started with a subcutaneous administration of the human menopausal gonadotropin (hMG, 300 IU/ day) on menstruation cycle-day 3 until approximately cycle-day 12. The hMG dosage was adjusted according to the ovarian response (i.e., ovarian follicles growth), which was assessed every two days using a transvaginal ultrasound. To prevent premature ovulation, in addition to the hMG, a subcutaneous dose of the gonadotropin releasing-hormone antagonist (GnRH, ~0.25mg/ml) was administered when at least one follicle had reached a diameter of 13-14 mm. Once at least one follicle had reached a diameter of 17mm or more, a subcutaneous dose of the human chorionic gonadotropin (HCG, 5000 IU) was administered for final maturation induction while the hMG and the GnRH antagonist were discontinued. Approximately 36 hours after the HCG administration, an ultrasound-guided ovarian puncture was performed to remove oocytes.

#### Minimal ovarian stimulation in vitro-fertilization - intervention

The MS-IVF treatment protocol started with taking an oral dose of letrozole 5mg/day on cycle-day 3 until cycle-day 7 when a subcutaneous dose of hMG (150 IU/day) is administered during 3 to 5 days in addition to letrozole. The hMG dosage was adjusted according to the ovarian response (i.e., ovarian follicles growth), which was assessed every two days using a transvaginal ultrasound. A subcutaneous dose of the gonadotropin releasing-hormone antagonist (GnRH, ~0.25mg/ml) was administered when at least one follicle had reached a diameter of 13-14 mm. Once at least one follicle had reached a diameter of 17mm or more, a subcutaneous dose of the human chorionic gonadotropin (HCG, 5000 IU) was administered for final maturation induction while the hMG and the GnRH antagonist were discontinued. Approximately 36 hours after the HCG administration, an ultrasound-guided ovarian puncture was performed to remove oocytes.

### Effect outcomes

#### Primary outcome

The primary outcome was the pregnancy rate per initiated cycle. To assess this outcome, women were asked to perform a beta-HCG blood test to confirm a pregnancy 12 days after the embryo was transferred in a laboratory of their preference. The pregnancy rate per initiated cycle was estimated by dividing the number of women with a positive beta-HCG by the total number of initiated cycles.

#### Secondary outcomes

Secondary outcomes included the duration of ovarian stimulation (i.e., number of stimulation days), cycle cancelation rate, the number of oocytes retrieved, the number of oocytes that reached maturation (i.e., metaphase II, [MII]), and the number and quality of embryos obtained from the IVF.

The number of days needed for ovarian stimulation was registered by the medical team. Cycle cancelation (i.e., absence of follicular growth after ovarian stimulation) was evaluated using transvaginal ultrasound by the medical team. The cycle cancelation rate is the number of cancelled cycles by the total number of initiated cycles. The number of oocytes retrieved by ultrasound-guided ovarian puncture, the number of oocytes that reached maturation MII, and the number and quality of embryos after IVF were evaluated by an embryologist who was blinded for the intervention. Three evaluations were performed at 16 - 18 hours, 25 - 27 hours, and 64 - 67 hours post-IVF. The score was composed by the Graduated Embryo Score (GES) criteria (Fisch *et al.*, 2001): nucleolar alignment along the pronuclear axis, regular cleavage and degree of fragmentation at the first cell division, and cell number and morphology. The maximum score is 100/ embryo. The total score was calculated by the sum of embryo scores. Higher scores indicate a better embryo quality. The medical team also registered whether the embryos obtained from IVF were transferred into the uterus or not.

### Cost outcome measures

In this economic evaluation, only medication costs (i.e., costs related to hMG and letrozole) were included in the analysis, because it was the main difference between the two treatment protocols. During follow-up, the total hMG dosage required for ovarian stimulation in both treatment protocols and the type of IVF (i.e., where sperm and oocytes are mixed and incubated overnight or ICSI) were registered by the medical team.

The price of hMG was €0.60 per international unit (IU) and that of letrozole 2.5mg was €8.19 per pill. The unit price of these medications was based on the average market price in 2017 and adjusted to 2019 using consumer price indices (The World Bank, n.d.). To allow for international comparison, the results were converted from R$ to Euros (€) based on 2019 Purchasing Power Parity (1International dollar 1U$$ = R$2.07 = €0.79) (OECD/Organisation for Economic Co-operation and Development, 2019).

### Other variables

At baseline, data were collected from the women’s medical records on age (years), body mass index (BMI, kg^.^m^2^), causes of infertility (i.e., unknown, endometriosis, diminished ovarian reserve, tubal factor, female plus male infertility, other causes), and blood levels of anti-Müllerian hormone (AMH, ng/mL).

### Statistical analysis

The unit of analysis was one cycle of IVF treatment, because information on the cost per cycle is probably most relevant for patients who need to pay for the treatment themselves (Souza, 2014). Descriptive statistics per treatment protocol group were performed at baseline. Continuous variables were described as means and standard deviations (SD), while categorical variables were described as absolute numbers and percentages.

### Effectiveness analysis

As this was a non-randomized study, statistical methods were needed to control for confounding by indication (Rosenbaum & Rubin, 1983; Austin, 2011). For this purpose, propensity score adjustment was used. The propensity score was estimated based on the women’s age, BMI, causes of infertility, AMH, and type of IVF using the *pscore* package in STATA 16SE (Becker & Ichino, 2002). Subsequently, primary and secondary outcomes were regressed upon a variable indicating their treatment protocol group and the estimated propensity score. Differences in effects between groups were presented as absolute mean differences. Uncertainty around the mean differences was presented using 95% confidence intervals (95%CIs). Some women underwent more than one cycle in the MS-IVF treatment protocol, while this was not the case in the C-IVF. Therefore, we could not use mixed-effect models, because the two-level structure of cycles clustered within women does not exist in one group compromising comparability (Wooldridge, 2008).

### Cost-effectiveness analysis

A cost-effectiveness analysis was performed for the primary effect outcome using STATA 16SE. Cost and effect differences between groups were estimated using seemingly unrelated regression adjusted for the propensity score, while simultaneously accounting for the correlation between costs and effects (Fiebig, 2008). Bias-corrected and accelerated bootstrapping with 5,000 replications was used to estimate the joint uncertainty surrounding differences in effects and costs between groups. Incremental Cost-Effectiveness Ratios (ICERs) were calculated by dividing the difference in costs by the difference in effects. Bootstrapped cost-effect pairs were plotted on cost-effectiveness planes (CE-plane) (Black, 1990). Cost-effectiveness acceptability curves (CEACs) were estimated, showing the probability of the MS-IVF being cost-effective compared to C-IVF for a range of willingness-to-pay thresholds (i.e., the maximum amount of money a patient is willing to pay for a positive pregnancy test) (Fenwick *et al.*, 2004).

### Sensitivity analysis

Sensitivity analyses (SA) were conducted to explore the robustness of the primary outcome analysis. SA1 considered the pregnancy rate per embryo transferred into the uterus as the effect outcome. The pregnancy rate per embryo transfer was estimated by dividing the number of positive pregnancy tests by the number of cycles in which embryos were transferred into the uterus. This additional outcome was chosen because some women do not respond to ovarian stimulation and, hence, the IVF is cancelled due to a lack of oocytes. SA2 did not include the propensity score adjustment.

## RESULTS

### Participants

**Figure 1:**
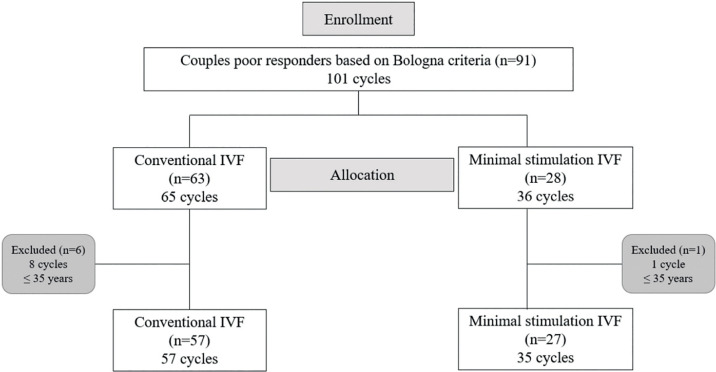
Flow diagram of enrollment, group allocation, and inclusion for the primary outcome IVF: *in vitro* fertilization; n: number of couples.

Out of the 227 couples who sought assisted reproduction treatments at the clinic during the study period, all 84 eligible couples agreed to participate and underwent a total of 92 IVF cycles during the10-month follow-up (C-IVF = 57 cycles (n=57) and MS-IVF = 35 (n=27) cycles) (Figure 1).

Two couples received both treatment protocols once. None of the couples underwent more than 1 cycle in the C-IVF treatment protocol, while in the MS-IVF, 3 couples underwent 3 cycles and 2 couples underwent 2 cycles resulting in 8 additional cycles. At baseline, no clinically relevant differences were found between the MS-IVF and the C-IVF treatment protocols (Table 1). Baseline and follow-up data were complete for all participants. In the C-IVF group, ICSI was used in 34 cycles while in the MS-IVF, ICSI was used in 19 cycles (Table 1).

**Table 1:** Characteristics of women in the groups.

	MS-IVF (n=35 cycles)	C-IVF (n=57 cycles)
Age, years, mean (SD)	41.2 (2.4)	40.4 (2.8)
BMI, kg.m^2^ mean (SD)	24.1 (2.9)	24.0 (2.7)
AMH, ng/mL mean (SD)	0.8 (0.9)	0.6 (0.5)
Primary cause of infertility, n (%) Unknown Endometriosis Diminished ovarian reserve Tubal factor Male infertility plus ovarian poor response Other causes	0 6 (17.1) 11 (31.4) 7 (20.0) 0 11 (31.4)	3 (5.3) 15 (26.3) 10 (17.5) 5 (8.8) 6 (10.5) 18 (31.6)
Type of IVF, n^§^ (%) Conventional ICSI	0 19 (54.3)	10 (17.6) 34 (59.6)

MS-IVF: minimal ovarian stimulation *in-vitro* fertilization. C-IVF: conventional ovarian stimulation *in-vitro* fertilization. BMI: body mass index. AMH: anti-Müllerian hormone. SD: standard deviation. ICSI: intracytoplasmic sperm injection.

§ corresponds to the number of cycles in which embryos were transferred into the uterus.

**Table 2:** Mean effects by group and mean differences at follow-up.

	MS-IVF (n=35 cycles)	C-IVF (n=57 cycles)	Difference (95%CI)	Adjusted difference (95% CI)
Pregnancy rate/initiated cycle, n (%)	1 (2.9)	7 (12.3)	-9.4% (-21.4; 2.6)	-5.1% (-13.2; 5.2)
Number of stimulation days (SE)	9.6 (0.5)	9.8 (0.3)	-0.2 (-1.3; 1.0)	-0.1 (-1.3; 1.1)
Cycle cancelation rate, n (%)	21 (60.0)	18 (31.6)	28.4% (8.0; 48.9)	18.8% (-0.6; 38.1)
Number of Oocytes retrieved (SE)	1.1 (0.2)	2.0 (0.2)	-0.9 (-1.6; -0.2)	-0.7 ^*^ (-1.4; -0.01)
Number of MII (SE)	1.0 (0.2)	1.8 (0.2)	-0.8 (-1.4; -0.1)	-0.5 (-1.2; 0.06)
Number of Embryos (SE)	0.5 (0.1)	1.3 (0.2)	-0.7 (-1.2; -0.2)	-0.6 ^*^ (-1.1; -0.1)
Embryos transfer, n (%)	14 (40.0)	39 (68.4)	-28.4% (-48.9; -0.1)	-18.8% ^*^ (-38.1; -0.6)
Quality of Embryos, points (SE)	88 .6 (14)	140 .4 (17)	-51.8 (-113.0;9.4)	-52.7 (-114.0; 9.0)
Pregnancy rate/ embryo transfer, n^§^ (%)	1 (7.1)	7 (17.9)	-10.8% (-33.4;11.8)	-8.8% (-23.6; 14.0)

SE: standard error. MII: oocytes that reached maturation metaphase II. NA: not applicable. Adjusted difference for the propensity score.

* Statistically significant. §corresponds to the number of cycles in which embryos were transferred into the uterus.

In the MS-IVF treatment protocol, 71% (n=25) of the cycles needed hMG dose adjustment (total adjusted dosage = 21459 UI) while such an adjustment was needed for 88% (n=50) of the cycles in the C-IVF (total adjusted dose =-36,450 UI). The total dosage of hMG required for ovarian stimulation was significantly lower in the MS-IVF treatment protocol compared to C-IVF (mean difference = -2223 IU; 95% CI, -2494 to -1953, supplementary Table 1).

**Supplementary Table 1:** Medication usage and costs by treatment protocol group.

	MS-IVF (n=35 cycles)	C-IVF (n=57 cycles)	Difference (95%CI)
Medication dosage, mean (SD)			
hMG	441 IU (490)	2665 IU (708)	-2223 IU (-2494, -1953)
Letrozole,	9.7 pill (43)	0	9.7 pill (9.3, 10.1)
Medication costs €, mean (SD)			
hMG	€266 (295)	€1607 (427)	€-1341 (-1504, -1177)
Letrozole	€80	0	€80 (76, 83)
Total medication costs	€346 (298)	€1607 (427)	€-1260 (-1401; -1118)

hMG: human menopausal gonadotropin. IU: international units. 1IU= €0.60. 1 pill = €8.19. €: euros.

### Effectiveness

There was no significant difference between groups in the pregnancy rate per initiated cycle (adjusted mean difference = -5.1%; 95%CI, -13.2 to 5.2, [Table 2]). The number of ovarian stimulation days, cycle cancelation rate, number of MII, and quality of embryos did not differ between groups, while the MS-IVF treatment protocol resulted in a significantly lower number of oocytes retrieved (adjusted mean difference = -0.7, 95%CI, -1.4 to -0.01), lower number of embryos (adjusted mean difference = -0.6, 95%CI, -1.1 to -0.1), and lower percentage of embryos transferred into the uterus (adjusted mean difference = -18.8, 95%CI, -38.1 to -0.6) (Table 2). There was no significant difference in the pregnancy rate per embryo transfer between groups either (adjusted mean difference -8.8%; 95%CI, -23.6 to 14.0).

### Costs

The mean medication cost of the additional 8 cycles in the MS-IVF group was €319 (95%CI, 147 to 492). Mean payer’s medication costs of MS-IVF were significantly lower compared to those of C-IVF (mean adjusted difference = €-1260; 95%CI, -1401 to -1118).

### Cost-effectiveness analysis

**Figure 2:**
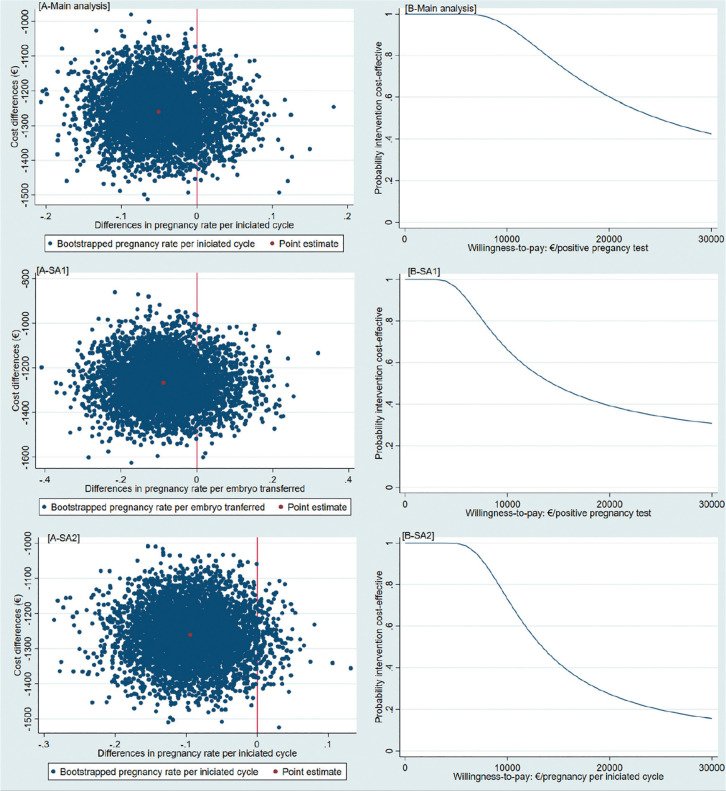
Cost-effectiveness planes [A] and Cost-effectiveness acceptability curves [B] [A-Main analysis] Cost-effectiveness plane indicating the uncertainty around the point estimate of the incremental cost-effectiveness ratio regarding pregnancy rate per initiated cycle [B-Main analysis] Cost-effectiveness acceptability curve indicating the probability of cost-effectiveness for different willingness-to-pay thresholds per a positive pregnancy test (initiated cycle) [A-SA1] Cost-effectiveness plane indicating the uncertainty around the point estimate of the incremental cost-effectiveness ratio regarding pregnancy rate per embryo transfer [B-SA1] Cost-effectiveness acceptability curve indicating the probability of cost-effectiveness for different willingness-to-pay thresholds per a positive pregnancy test (embryo transfer) [A-SA2] Cost-effectiveness plane indicating the uncertainty around the point estimate of the incremental cost-effectiveness ratio regarding pregnancy rate per initiated cycle unadjusted [B-SA2] Cost-effectiveness acceptability curve indicating the probability of cost-effectiveness for different willingness-to-pay thresholds per a positive pregnancy test (initiated cycle unadjusted for baseline characteristics) €: euros

For the primary outcome, the MS-IVF treatment protocol was on average significantly less costly than C-IVF, but also less effective albeit not significantly. As a consequence, most of the bootstrapped cost-effective pairs (85%) were located in the south-west quadrant (Table 3, Figure 2).

**Table 3:** Results of the cost-effectiveness analysis.

Effect Outcome	Costs difference, € (95% CI)	Effect difference (95% CI)	ICER €/ effect gained	Distribution on the Cost-Effectiveness plane			
				North-East	South-East	South-West	North-West
**Main Analysis**							
Pregnancy rate/ initiated cycle	-1260 (-1401; -1118)	-0.051 (-0.132; 0.052)	24735	0%	15%	85%	0%
**SA1**							
Pregnancy rate/ embryo transfer	-1266 (-1450; -1038)	-0.088 (-0.236; 0.140)	14341	0%	17%	82%	0%
**SA2**							
Main analysis unadjusted	-1261 (-1404; -1103)	-0.094 (-0.197; 0.002)	13381	0%	3%	97%	0%

SA1: sensitivity analysis 1, the pregnancy rate per embryo transfer as primary effect outcome (i.e., the number of cycles in which embryos were transferred into the uterus were included in the denominator). SA2: sensitivity analysis 2, main analysis not adjusted for the propensity score.

The CEAC shows that the probability of the MS-IVF treatment protocol being cost-effective compared to the C-IVF was 1.00 at a willingness-to-pay of €0 per established pregnancy. At willingness-to-pay thresholds of €5,000, €10,000, €15,000, and €20,000 per established pregnancy the probabilities of MS-IVF being cost-effective compared to C-IVF were 0.99, 0.94, 0.76, and 0.60, respectively (Figure 2).

### Sensitivity analysis

The results of both sensitivity analyses were similar to the main analysis (Table 3, Figure 2). For the pregnancy rate per embryo transfer outcome (SA1), there was no significant difference between groups (adjusted mean difference = -8.8%; 95%CI, -23.6 to 14.0). In the cost-effectiveness analysis, most bootstrapped cost-effect pairs were in the south-west quadrant of the CE-plane (82%, Table 3). The probabilities of cost-effectiveness of the intervention compared to control was 0.96, 0.66, 0.48, and 0.39 at willingness-to-pay of €5,000, €10,000, €15,000 and €20,000 per established pregnancy, respectively.

Without adjustment for the propensity score (SA2), there was no significant difference in pregnancy rates between groups either (unadjusted mean difference = -9.4%; 95%CI 19.7 to 0.2). The proportion of the bootstrapped cost-effect pairs in the south-west quadrant increased from 85% (i.e., main analysis) to 97%. As a consequence, the probabilities of cost-effectiveness decreased as willingness-to-pay threshold increase. For instance, the probability of cost-effectiveness decreased from 0.94 (i.e., main analysis) to 0.73 at a willingness-to-pay of €10,000 per established pregnancy and from 0.76 (i.e., main analysis) to 0.42 at a €15,000 per established pregnancy.

## DISCUSSION

### Main findings

This study evaluated the effectiveness and cost-effectiveness of the MS-IVF treatment protocol compared to C-IVF in women with poor response to ovarian stimulation. The results showed that there was no statistically significant difference between groups in the pregnancy rate per initiated cycle, number of stimulation days, cancelation rate, number of MII, and quality of embryos while there was significantly lower number of oocytes retrieved and number of embryos in the MS-IVF compared to the C-IVF. The MS-IVF treatment protocol was on average less costly and less effective compared to C-IVF. Because of the cost savings associated with MS-IVF, the probability of MS-IVF being cost-effective compared to C-IVF was high and ranged from 1 to 0.76 for willingness-to-pay thresholds of €0 to €15,000 per established pregnancy and dropped to 0.60 at a ceiling ratio of €20,000.

### Comparison with other effectiveness studies

Similar to our results, a systematic review and meta-analysis that evaluated the effectiveness of low dosing of gonadotropins (e.g., hMG) combined with oral compounds (e.g., letrozole and clomiphene) compared with high doses of gonadotropins in poor responders showed no statistical difference in pregnancy rates between groups (meta-analysis based on 3 randomized clinical trials: risk rate (RR) 0.90, 95% CI, 0.63 to 1.27) (Youssef *et al.*, 2018).

Regarding the secondary outcomes of this study, the aforementioned systematic review and meta-analysis found significant lower ovarian stimulation days and significantly more treatment cycles were cancelled due to poor ovarian response in women who underwent MS-IVF than in women who underwent C-IVF, while we found no significant difference (Youssef *et al.*, 2018). They also found no significant differences between groups in the number of oocytes retrieved, the number of MII oocytes, and the number of embryos obtained, while we found significant lower numbers of oocytes retrieved and embryos for the MS-IVF treatment protocol compared to the C-IVF (Youssef *et al.*, 2018). These differences might be related to differences in medication dosages and other differences in populations, such as the underlying causes of infertility. This needs to be evaluated in future research.

### Comparison with other cost-effectiveness studies

Until now, relatively few studies assessed the cost-effectiveness of MS-IVF compared to C-IVF. We only found three of such studies, including two in a European population (i.e., Dutch and Bulgarian) and one in a US population (Polinder *et al.*, 2008; Crawford *et al.*, 2016; Benbassat *et al.*, 2017). Comparing our results with those of the previous studies is limited, because the latter had less strict inclusion criteria (e.g., including not only poor responders), a different design (e.g., trial-based and model based economic evaluations), different perspectives (e.g., costs related to sick leave, obstetric and post-natal costs of live births), and different outcomes (e.g., pregnancy within 1 year leading to term live birth). However, one study found no difference in pregnancy rate between the two treatment protocols (Polinder *et al.*, 2008), while the other two studies showed that the MS-IVF was more effective than C-IVF (Crawford *et al.*, 2016; Benbassat *et al.*, 2017). Overall, our findings are in line with their conclusions, namely that MS-IVF is more likely to be cost-effective than C-IVF.

### Impact on decision making

Currently, assisted reproduction treatments are mainly provided by private institutions and health insurance does not cover infertility treatments in Brazil (Zegers-Hochschild *et al.*, 2019). Therefore, only a small proportion of infertile couples can afford IVF treatment protocols in Brazil (Zegers-Hochschild *et al.*, 2019). In addition, research has shown that assisted reproduction treatments are more costly for women aged 40 or over compared with younger women because success rates are lower (Chambers *et al.*, 2006). This has led to a debate about the viability of using private and public funds for treating women with low chances of pregnancy success worldwide (Griffiths *et al.*, 2010; Dyer *et al.*, 2013; Stephen *et al.*, 2016; Benbassat *et al.*, 2017) and in Brazil in particular (Souza, 2014; Corrêa & Loyola, 2015). Our finding that MS-IVF is likely to be cost-saving compared to C-IVF in poor responders may add to this debate. Also, the cost savings associated with MS-IVF compared with C-IVF may enable couples to undergo more treatment cycles if they should pay for them themselves.

### Strengths and limitations

This study has a number of strengths. First, analyses were based on routinely collected (i.e., real world) data. Real world data studies assess health outcomes and costs in routine clinical practice, thereby increasing generalizability (Berger *et al.*, 2017). Second, this study is the first to provide information on the effectiveness and cost-effectiveness of assisted reproduction treatments among poor responders in Brazil. This information is important, because couples pay per cycle themselves and getting pregnant is their primary aim. To the best of our knowledge, a similar economic evaluation has not been performed in Brazil to aid couple-clinician’s decision making. Third, we used propensity scores to correct for baseline imbalances, bootstrapping to deal with skewed costs, and used seemingly unrelated regression (or bivariate regression) to preserve the correlation between costs and effects. These methods can all be considered the current state-of-art (van Dongen *et al.*, 2020). This is important, because a previous review indicated that statistical quality of trial-based economic evaluations in the field of obstetrics and gynaecology is typically sub-optimal (El Alili *et al.*, 2017).

This study also has some limitations. First, due to the non-randomized nature of the study the possibility for making causal inferences about the effect of the intervention is limited. To overcome this limitation, we used propensity score adjustment, which improves the comparability of the groups and as such decreases the bias introduced by confounding by indication that is inherent from using observational data (Rosenbaum & Rubin, 1983; Austin, 2011). Second, two couples received both treatment protocols meaning that observations between groups were not independent for those cases. Given the relatively small number of such cases we do not think the results would be biased. Third, costs included only the costs of the ovarian stimulation protocols, whereas guidelines recommend the use of broader perspectives, such as a healthcare perspective (e.g. Brazilian guideline) (Ministério da Saúde, 2014). As a consequence of the restricted follow-up period, we did not include costs of pregnancy and delivery. This may lead to a considerable underestimation of total treatment costs because there may be substantial costs associated with obstetric care, especially if a multiple pregnancy is established. However, medication costs are most likely to be of interest to patients, who typically have to pay themselves for assisted reproduction treatments in Brazil. Fourth, we were not able to follow-up on the ongoing pregnancies, so we could not compare the difference in costs between MS-IVF and C-IVF with the difference in live birth rates. If MS-IVF results in less live birth pregnancies per cycle, it may in the end be even more expensive than C-IVF, because more cycles will then be needed for a live birth. However, some of these costs may be off-set by lower obstetric care costs in the MS-IVF group and previous studies suggest that there are no differences in live births between low doses and high doses of gonadotropins (Youssef *et al.*, 2018). Research is, therefore, needed to investigate the long-term impact of the different IVF treatment protocols on longer-term outcomes, such as live births and also costs related to obstetric care. Fifth, health-related quality of life, patient distress, or side effects for the different treatment protocols were not evaluated either. It is known that the stress arising from the use of injectable medications and the procedures (e.g., ovarian puncture and transvaginal ultrasound) that are part of IVF treatment protocols can lead to more general psychological stress. Although, evidence suggests that the MS-IVF may reduce drop-out due to physical and psychological issues compared to the conventional protocol (Verberg *et al.*, 2008). Future research is, therefore, advised to include these outcomes in the analysis. Sixth, it is unknown whether the current findings are generalizable to other regions in Brazil and other countries, because this study only included a population from one clinic in a Southern city of Brazil. Future investigation of the cost-effectiveness of IVF treatment protocols should include a representative sample of all poor responders to ovarian stimulation in Brazil and worldwide.

## CONCLUSIONS

This study evaluated the effectiveness and cost-effectiveness of the MS-IVF treatment protocol compared to C-IVF in women with poor response to controlled ovarian hyperstimulation. The results showed that there was no significant difference between groups in the pregnancy rate per initiated cycle. Due to the cost savings associated with MS-IVF, the probability of MS-IVF being cost-effective compared to C-IVF was high (1.00 at a ceiling ratio of €0 per established pregnancy). However, because MS-IVF is on average less effective than C-IVF, albeit non-significantly, the decision about which treatment protocol to use should be based on the preferences of the couples and the expert medical doctors. Future research is needed to investigate the long-term impact of the IVF treatment protocols on term live births, health-related quality of life, as well as the costs related to obstetric care in populations in other regions of Brazil and other countries.

## References

[r1] Austin PC. (2011). An Introduction to Propensity Score Methods for Reducing the
Effects of Confounding in Observational Studies. Multivariate Behav Res.

[r2] Becker SO, Ichino A. (2002). Estimation of Average Treatment Effects Based on Propensity
Scores. Stata J.

[r3] Benbassat B, Mitov K, Savova A, Tachkov K, Petrova G. (2017). Cost-effectiveness of different types of COH protocols for in
vitro fertilization at national level. Biotechnol Biotechnol Equip.

[r4] Berger ML, Sox H, Willke RJ, Brixner DL, Eichler HG, Goettsch W, Madigan D, Makady A, Schneeweiss S, Tarricone R, Wang SV, Watkins J, Mullins CD. (2017). Good Practices for Real-World Data Studies of Treatment and/or
Comparative Effectiveness: Recommendations from the Joint ISPOR-ISPE Special
Task Force on Real-World Evidence in Health Care Decision
Making. Value Health.

[r5] Black WC. (1990). The CE plane: a graphic representation of
cost-effectiveness. Med Decis Making.

[r6] Brasil (2014). Diretrizes metodológicas: Diretriz de Avaliação
Econômica / Ministério da Saúde, Secretaria de
Ciência, Tecnologia e Insumos Estratégicos, Departamento de
Ciência e Tecnologia.

[r7] Chambers GM, Ho MT, Sullivan EA. (2006). Assisted reproductive technology treatment costs of a live birth:
an age-stratified cost-outcome study of treatment in
Australia. Med J Aust.

[r8] Corrêa MCDV, Loyola MA. (2015). Assisted reproductive technologies in Brazil: options to improve
access. Physis.

[r9] Crawford NM, Sahay KM, Mersereau JE. (2016). Mild Stimulation versus Conventional IVF: A Cost-Effectiveness
Evaluation. Open J Obstet Gynecol.

[r10] Drummond MF, Stoddart GL, Torrance GW. (2005). Oxford.

[r11] Duarte-Filho OB, Bianchi PHM, Lobel ALS, Peregrino PFM, Piccinato CA, Podgaec S. (2019). Assisted Reproductive Technologies in Latin America and Europe: a
Comparative Analysis of Reported Databases for 2013. Rev Bras Ginecol Obstet.

[r12] Dyer SJ, Patel M. (2012). The economic impact of infertility on women in developing
countries a systematic review. Facts Views Vis Obgyn.

[r13] Dyer SJ, Sherwood K, McIntyre D, Ataguba JE. (2013). Catastrophic payment for assisted reproduction techniques with
conventional ovarian stimulation in the public health sector of South
Africa: frequency and coping strategies. Hum Reprod.

[r14] Eijkemans MJ, Heijnen EM, de Klerk C, Habbema JD, Fauser BC (2006). Comparison of different treatment strategies in IVF with
cumulative live birth over a given period of time as the primary end-point:
methodological considerations on a randomized controlled non-inferiority
trial. Hum Reprod.

[r15] El Alili M, van Dongen JM, Huirne JAF, van Tulder MW, Bosmans JE (2017). Reporting and Analysis of Trial-Based Cost-Effectiveness
Evaluations in Obstetrics and Gynaecology. Pharmacoeconomics.

[r16] Farquhar C, Marjoribanks J. (2018). Assisted reproductive technology: an overview of Cochrane
Reviews. Cochrane Database Syst Rev.

[r17] Fenwick E, O’Brien BJ, Briggs A. (2004). Cost-effectiveness acceptability curves--facts, fallacies and
frequently asked questions. Health Econ.

[r18] Ferraretti AP, La Marca A, Fauser BC, Tarlatzis B, Nargund G, Gianaroli L, ESHRE working group on Poor Ovarian Response Definition (2011). ESHRE consensus on the definition of ‘poor response’ to ovarian
stimulation for in vitro fertilization: the Bologna criteria. Hum Reprod.

[r19] Fiebig DG., Baltagi BH (2008). A Companion to Theoretical Econometrics.

[r20] Fisch JD, Rodriguez H, Ross R, Overby G, Sher G. (2001). The Graduated Embryo Score (GES) predicts blastocyst formation
and pregnancy rate from cleavage-stage embryos. Hum Reprod.

[r21] Griffiths A, Dyer SM, Lord SJ, Pardy C, Fraser IS, Eckermann S (2010). A cost-effectiveness analysis of in-vitro fertilization by maternal
age and number of treatment attempts. Hum Reprod.

[r22] Husereau D, Drummond M, Petrou S, Carswell C, Moher D, Greenberg D, Augustovski F, Briggs AH, Mauskopf J, Loder E, CHEERS Task Force (2013). Consolidated Health Economic Evaluation Reporting Standards
(CHEERS) statement. Value Health.

[r23] Nargund G, Datta AK, Fauser BCJM. (2017). Mild stimulation for in vitro fertilization. Fertil Steril.

[r24] Nargund G, Frydman R. (2007). Towards a more physiological approach to IVF. Reprod Biomed Online.

[r25] OECD: Organisation for Economic Co-operation and
Development (2019). Conversion rates - Purchasing power parities (PPP) - OECD
Data. The OECD [Internet].

[r26] Oehninger S. (2011). Poor responders in in vitro fertilization (IVF) therapy: the
challenge continues. Facts Views Vis Obgyn.

[r27] Oudendijk JF, Yarde F, Eijkemans MJ, Broekmans FJ, Broer SL. (2012). The poor responder in IVF: is the prognosis always poor?: a
systematic review. Hum Reprod Update.

[r28] Polinder S, Heijnen EM, Macklon NS, Habbema JD, Fauser BJ, Eijkemans MJ. (2008). Cost-effectiveness of a mild compared with a standard strategy
for IVF: a randomized comparison using cumulative term live birth as the
primary endpoint. Hum Reprod.

[r29] Rosenbaum PR, Rubin DB. (1983). The central role of the propensity score in observational studies
for causal effects. Biometrika.

[r30] Schmidt L, Sobotka T, Bentzen JG, Nyboe Andersen A, ESHRE Reproduction and Society Task Force (2012). Demographic and medical consequences of the postponement of
parenthood. Hum Reprod Update.

[r31] Shrestha D, La X, Feng HL. (2015). Comparison of different stimulation protocols used in in vitro
fertilization: a review. Ann Transl Med.

[r32] Song Y, Li Z, Wu X, Wang X, Xiao J, Wang B. (2014). Effectiveness of the antagonist/letrozole protocol for treating
poor responders undergoing in vitro fertilization/intracytoplasmic sperm
injection: a systematic review and meta-analysis. Gynecol Endocrinol.

[r33] Souza MCB. (2014). Latin America and access to Assisted Reproductive Techniques: A
Brazilian perspective. JBRA Assist Reprod.

[r34] Stephen EH, Chandra A, King RB. (2016). Supply of and demand for assisted reproductive technologies in
the United States: clinic- and population-based data,
1995-2010. Fertil Steril.

[r35] Sun H, Gong TT, Jiang YT, Zhang S, Zhao YH, Wu QJ. (2019). Global, regional, and national prevalence and disability-adjusted
life-years for infertility in 195 countries and territories, 1990-2017:
results from a global burden of disease study, 2017. Aging (Albany NY).

[r36] The World Bank [Internet] (2021). Inflation, consumer prices (annual %) - Brazil.

[r37] van Dongen JM, El Alili M, Varga AN, Guevara Morel AE, Jornada Ben A, Khorrami M, van Tulder MW, Bosmans JE (2020). What do national pharmacoeconomic guidelines recommend regarding
the statistical analysis of trial-based economic
evaluations?. Expert Rev Pharmacoecon Outcomes Res.

[r38] Verberg MF, Eijkemans MJ, Heijnen EM, Broekmans FJ, de Klerk C, Fauser BC, Macklon NS (2008). Why do couples drop-out from IVF treatment? A prospective cohort
study. Hum Reprod.

[r39] Verberg MF, Eijkemans MJ, Macklon NS, Heijnen EM, Baart EB, Hohmann FP, Fauser BC, Broekmans FJ. (2009). The clinical significance of the retrieval of a low number of
oocytes following mild ovarian stimulation for IVF: a
meta-analysis. Hum Reprod Update.

[r40] Wooldridge JM. (2008). Introductory Econometrics: A Modern Approach.

[r41] Youssef MA, van Wely M, Mochtar M, Fouda UM, Eldaly A, El Abidin EZ, Elhalwagy A, Mageed Abdallah AA, Zaki SS, Abdel Ghafar MS, Mohesen MN, van der Veen F (2018). Low dosing of gonadotropins in in vitro fertilization cycles for
women with poor ovarian reserve: systematic review and
meta-analysis. Fertil Steril.

[r42] Zegers-Hochschild F, Schwarze JE, Crosby JA, Musri C, Urbina MT. (2019). Assisted reproductive techniques in Latin America: The Latin
American registry, 2016. JBRA Assist Reprod.

[r43] Zhang Y, Zhang C, Shu J, Guo J, Chang HM, Leung PCK, Sheng JZ, Huang H. (2020). Adjuvant treatment strategies in ovarian stimulation for poor
responders undergoing IVF: a systematic review and network
meta-analysis. Hum Reprod Update.

